# Commentary: Safety and feasibility of CRISPR-edited T cells in patients with refractory non-small-cell lung cancer

**DOI:** 10.3389/fonc.2020.01726

**Published:** 2020-09-10

**Authors:** Rafael Rosell, Martyna Filipska, Imane Chaib, David Lligé, Fernando Laguia

**Affiliations:** Germans Trias i Pujol Research Institute and Hospital, Badalona, Spain

**Keywords:** PD-1, PD-L1, T Cells, CRISPR, PI3K, Akt, MAPK, NSCLC

## Introduction

CRISPR/Cas9 ribonucleoprotein-mediated editing has been used to disrupt the *PDCD1* gene encoding programmed cell death-1 (PD-1) in human T cells, resulting in a significantly reduced PD-1 expression without affecting T cell viability during prolonged *in vitro* culture. Modified T-cells show up-regulation of IFN-gamma production and enhanced cytotoxicity (1) and also augmented chimeric antigen receptor (CAR) T cell cytokine production and cytotoxicity toward PD-L1-expressing cancer cells *in vitro* (2). Moreover, genome-wide CRISPR-Cas9 screening has served to identify novel therapeutic targets. For example, the mRNA decapping enzyme scavenger (DCPS) gene, which is essential for acute myeloid leukemia (AML) survival, thus allowing treatment of AML with RG3039, a DCPS inhibitor used to treat spinal muscular atrophy (3). Similarly, CRISPR-Cas9 genome-wide screening in multiple myeloma cells identifies mechanisms whereby CSN9 signalosome subunits modulate cereblon expression levels and sensitivity to immunomodulatory drugs, i.e., lenalidomine and pomalidomide (4). A CRISPR-assisted telomerase-activating gene expression system, using Cas9 as an effector gene, killed various cancer cell lines, including A549, PANC-1, and others, without affecting normal cells (5). Other genome-wide CRISPR screening in CD8 T cells in the context of immunotherapy have identified a RNA Helicase *Dhx37* that modulates NF-kB function and suppresses T cell production in breast cancer. Targeting DHX37 is suggested could be of significant clinical relevance (6)

## Subject

A phase I clinical trial of CRISPR-Cas9 PD-1-edited T cells in non-small-cell lung cancer demonstrates that the disruption of PD-1 on T cells generally induced grade 1 or 2 adverse events, and the most frequent events were lymphopenia, fatigue, leukopenia, fever, arthralgia, and skin rash. Flow cytometric analysis demonstrated decreased PD-1 expression, with median disruption of 46.3% in edited T cells. NGS targeted sequencing of the PD-1 gene indicated that the median editing efficiency of all 12 patients was 5.8%. A significant increase in the proportion of CD8^+^IFN-gamma^+^ cells was found in edited T cells (7).

## Discussion

### Is CRISPR-Cas9 -Mediated PD-1 Disruption a Solution in Non-small-Cell Lung Cancer?

**Figure 1:**
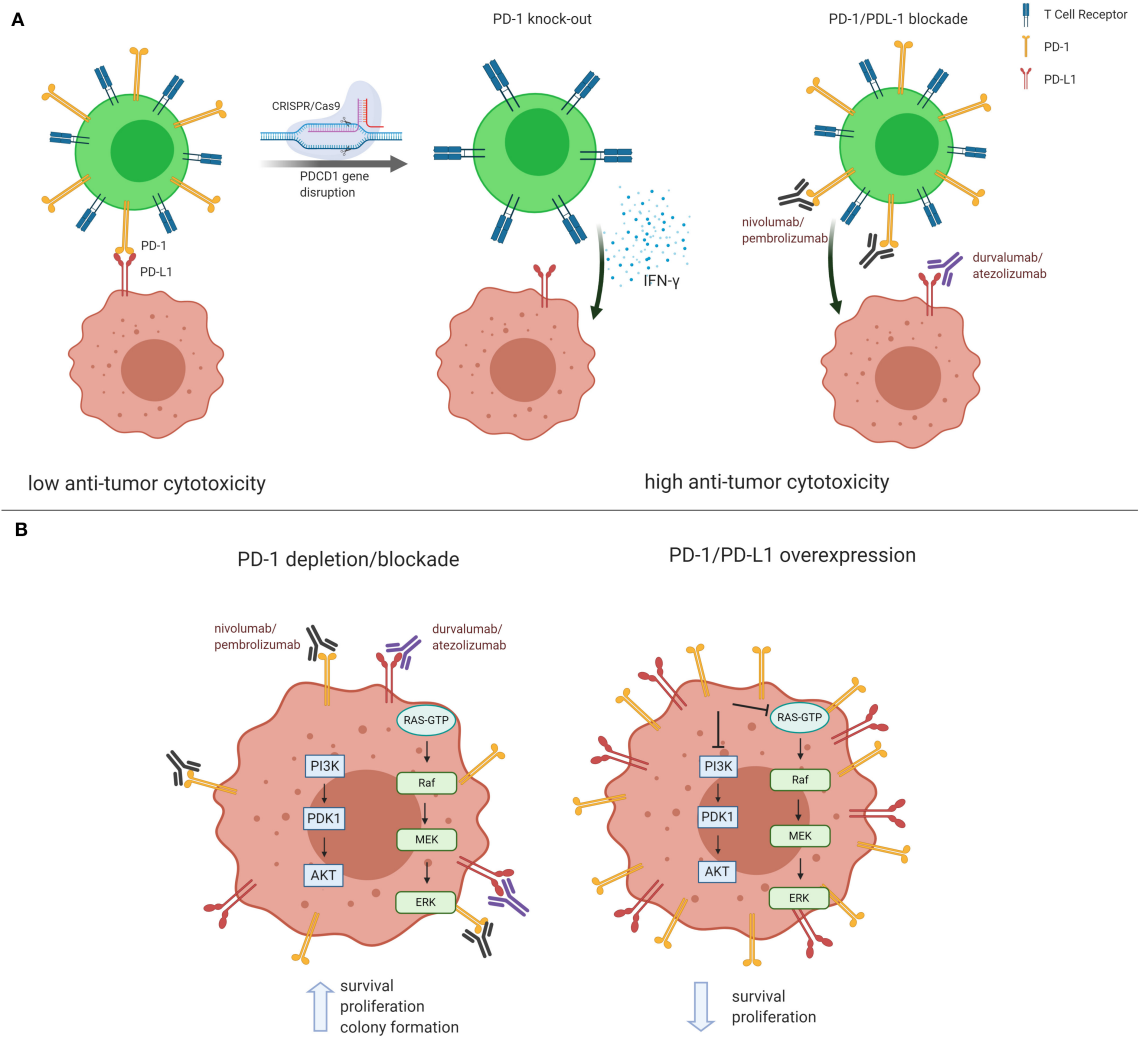
Conflicting role of PD-1 and PD-L1 in cancer management. **(A)** In the classical understanding of PD-1/PD-L1 regulation, PD-1 expressed on mature cytotoxic T lymphocytes reduces anti-tumor cytotoxicity via binding to tumor PD-L1. Both PD-1/PD-L1 CRISPR/Cas9-mediated PD-1 knockdown and blockade in T cells result in higher anti-tumor cytotoxicity. **(B)** Recent reports show that PD-1/PD-L1 blockade and/or silencing lead to PI3K/AKT and MEK/ERK1/2 pathway activation, thus inducing tumor growth and survival. The opposite effect is observed when PD-1/PD-L1 are found overexpressed in cancer cells. Figure created using BioRender.

PD-1 receptor is mainly expressed on mature cytotoxic T lymphocytes in peripheral tissues and the tumor microenvironment. PD-1 expression is inducible upon the activation of T cells, and PD-1 acts as a coinhibitory receptor that functions as immune checkpoint to maintain the peripheral immune tolerance and prevents autoimmunity (8). PD-1 ligation by PD-L1 expressed on tumors cells transduces signaling via the immunoreceptor tyrosine-based inhibitory motif (ITIM) and the immunoreceptor tyrosine-based inhibitor switch motif (ITISM) of the PD-1 cytoplasmic tail, which further inhibits the PI3K/AKT, MAPK/ERK1/2, and/or mTOR, thus suppressing tumor growth (9). A new discovery shows that *PDCD1* knockdown in non-small-cell lung cancer cell lines, NCi-H1299 and Calu-1, reduced the mRNA and protein levels in PD-1 depleted cells compared to control cells. Moreover, *PDCD1* silencing resulted in increased cell proliferation and colony formation. After *PDCD1* knockdown, phospho(p)-AKT and pERK1/2 levels were increased in both cell lines. Over-expressing PDCD1 increased mRNA and protein levels, inhibited cell proliferation and colony formation in NCI-H1299 and Calu-1 cells with decreased p-AKT and p-ERK1/2 levels (10). A non-small-cell lung cancer patient having high PD-1 expression in the tumor biopsy had rapid progression to anti-PD-1 therapy. The finding was validated with the detection of PD-1 transcript in lung cancer cells in resected lung cancer tissues. Knockout or antibody blockade of PD-1 enhanced M109 (mouse lung cancer cell line) viability *in vitro*. Also, PD-1 blockade accelerated growth of M109-xenograft tumors (11). Immunoblot analysis disclosed that PD-1 is expressed by NSCLC cell lines and that the expressed PD-1 was 55 KDa in size, similar to the size of T-cell expressed PD-1. Furthermore, flow cytometry showed that PD-1 is expressed in a sub-population of all examined cancer cells (10). Simultaneous overexpression of both *PDCD1* and P*DCD1LG1*, which encodes PD-L1, significantly decreased cell proliferation, P-AKT, and p-ERK levels compared to cells transfected with *PDCD1*, P*DCD1LG1*, or the control (10). The treatment of Calu-1, SW480, HT-29 and other cell lines with PD-1-targeted nivolumab, or pembrolizumab, increased cell proliferation and higher p-AKT and p-ERK levels than the cells treated with an isotype control antibody. The same observations were seen in xenografted cells in mice (10). The aforementioned results raise doubts about the adequacy of CRISPR-Cas9-mediated-PD-1 disruption in T cells (1, 7). The clinical consequences of Wang et al. (10) are unknown at this moment, however it offers a new view that the tumor cell intrinsic PD-1/PD-L1 axis suppresses tumor growth and inhibits AKT and ERK1/2 signaling pathways and could prevent the interaction with PD-1 expressing cells. As in cancer cells, PD-1 also regulates PI3K/AKT, MAPK/ERK1/2 and mammalian target of rapamycin (mTOR) pathways in T cells (9). Further understanding of the tumor-suppressor function of cancer cell PD-1 receptor expression can provide a new perspective in cancer management. Treatment of lung cancer is currently based on immunotherapy with antibodies against PD-1 or PD-L1, alone or in combination with chemotherapy. The use of pembrolizumab alone, or in combination with chemotherapy, as well as atezolizumab plus chemotherapy, is recommended for stage IV non-small-cell lung cancer patients without driver alterations (EGFR or ALK) according to the American Society of Clinical Oncology and Ontario Health joint guideline (12). Tumor PD-L1 expression by immunohistochemistry is considered the standard practice. Response rate is low, with median progression free survival, that is generally short-lived (12). It is possible, as above described, that if tumor PD-1 is in equilibrium with tumor PD-L1, the disruption of intratumoral PD1-PD-L1 induced by monoclonal antibodies that block PD-1 (nivolumab or pembrolizumab) or PD-L1 (atezolizumab, durvalumab and avelumab) can provoke tumor hyper-progression which has been reported in several studies (13) (Figure 1). The figure summarizes the previous reports on the conflicting role that PD-1 or PD-L1 monoclonal antibodies could have in causing tumor progression. A phase 1 clinical trial of multiplex CRISPR-Cas9 editing to engineer T cells in three cancer patients was reported. Two genes encoding the endogenous T cell receptor (TCR) chains, *TRAC* and *TRBC*, were deleted in T cells to reduce TCR mispairing and to enhance the expression of a synthetic, cancer specific TCR transgene (NY-ESO-1). *PDCD1* was also removed. Modified T cells persisted for up to 9 months, suggesting the feasibility of CRISPR gene editing for cancer immunotherapy (14). The preliminary results are enticing and shed light on the plausibility that multiplex CRISPR-Cas9 editing to engineer T cells could be more effective in cancer patients.

In our opinion, in addition to assessing patients for PD-L1 expression in the tumor, the expression of PD-1 in the tumor could serve as a potential biomarker to define efficacy or tumor progression following immunotherapy with either anti-PD-1 o anti-PD-L1 monoclonal antibodies (13).

## Author Contributions

All authors listed have made a substantial, direct and intellectual contribution to the work, and approved it for publication.

## Conflict of Interest

The authors declare that the research was conducted in the absence of any commercial or financial relationships that could be construed as a potential conflict of interest.
